# Comparison of Synthetic Media Designed for Expansion of Adipose-Derived Mesenchymal Stromal Cells

**DOI:** 10.3390/biomedicines6020054

**Published:** 2018-05-14

**Authors:** Michelle Lensch, Angela Muise, Lisa White, Michael Badowski, David Harris

**Affiliations:** Biorepository, University of Arizona, AHSC 6122, P.O. Box 245221, Tucson, AZ 85724, USA; mlensch1@email.arizona.edu (M.L.); muisea@email.arizona.edu (A.M.); lisamwhite@email.arizona.edu (L.W.); badowski@email.arizona.edu (M.B.)

**Keywords:** stem cells, MSC, tissue engineering, regenerative medicine, expansion, synthetic media

## Abstract

Mesenchymal stromal cells (MSCs) are multipotent cells that can differentiate into various cell types, such as osteoblasts, myocytes, and adipocytes. This characteristic makes the cells a useful tool in developing new therapies for a number of common maladies and diseases. The utilization of animal-derived growth serum, such as fetal bovine serum (FBS), for the expansion of MSCs has traditionally been used for cell culture. However, in clinical applications, animal-derived products present limitations and safety concerns for the recipient, as exposure to animal (xeno-) antigens and infectious agents is possible. Multiple synthetic, xeno-free media have been developed to combat these limitations of animal-derived growth serum and have the potential to be used in ex vivo MSC expansion for clinical use. The goal of this study was to determine if xeno-free media are adequate to significantly and efficiently expand MSCs derived from adipose tissue. MSCs were cultured in both standard FBS-containing as well as xeno-free media. The media were compared for cell yield, viability, and phenotypic expression via flow cytometry and directed differentiation. The xeno-free media that were tested were StemMACS MSC Expansion Media (Miltenyi Biotec, Bergisch Gladbach, Germany), PLTMax Human Platelet Lysate (Sigma-Aldrich, St. Louis, MO, USA), and MesenCult-hPL media (Stemcell Technologies, Vancouver, BC, Canada). All xeno-free media showed promise as a feasible replacement for animal-derived growth serums. The xeno-free media expanded MSCs more quickly than the FBS-containing medium and also showed great similarity in cell viability and phenotypic expression. In fact, each xeno-free media produced a greater viable cell yield than the standard FBS-containing medium.

## 1. Introduction

Stem cell-based therapies can provide an alternative approach for repair and regeneration of defective tissues and organs and could revolutionize the field of regenerative medicine. Theoretically, induced pluripotent stem (iPS) cells and embryonic stem (ES) cells seem to be more useful for cell-based therapies, as they are immortal and highly proliferative. However, ethical considerations, legal regulations, and genetic manipulations limit their use for cell-based therapies. Mesenchymal stromal cells (MSCs) are non-hematopoietic multipotent stem cells that hold great promise for regenerative medicine. In the past decade, much research has been devoted to bone marrow-derived MSCs (BM-MSCs). Many clinical and preclinical studies have shown that these cells can be successfully employed for the treatment of various diseases and disorders [1]. Studies have shown differentiation into mesenchymal and non-mesenchymal cell types, including adipocytes, osteoblasts, myocytes, muscle, and neurons [2]. However, BM-MSCs are not always preferred because of the painful isolation process, low cell yield, and adult age of the cells. Thus, a search for alternative sources of MSCs is an important aspect to consider for present regenerative medicine applications.

Human adipose tissue may provide an alternative source of MSCs for bioengineering and regeneration of tissues. It can serve as a convenient and abundant source of stem cells with little discomfort for the patient, as the liposuction procedure is less invasive than bone marrow aspiration. Moreover, adipose tissue has 500-fold more stem cells than bone marrow per gram of tissue [3,4]. Millions of such cells can easily be obtained from a single individual, as large adipose samples can be obtained from multiple harvest sites, and the cells proliferate rapidly in vitro and have low levels of senescence after months of in vitro culture [5].

The purpose of the present study was to compare commercially available synthetic, xeno-free media designed for (bone marrow) MSC expansion. Specifically, we analyzed the ability of adipose-derived MSCs to grow, remain viable, and retain a typical MSC phenotype when placed in ex vivo cell culture for up to 5 weeks. All results were compared to MSCs grown in standard fetal bovine serum (FBS)-supplemented medium. In summary, each of the xeno-free media was superior to FBS-supplemented medium in terms of MSC growth, while all media preserved viable cell numbers and function. The MSC phenotype varied between the different media with time in culture, although an overall MSC phenotype was observed for each medium used in the MSC expansion.

## 2. Methods

### 2.1. Collection of Adipose Tissue

Adipose tissue was harvested from patients (all donors were healthy females between 40 years and 60 years of age) using a 2.4 mm cannula during a scheduled liposuction procedure, or with a handheld 10cc syringe during a cosmetic procedure. All samples were obtained along with written consent from the donors according to the instructions from the local Institution Review Board (IRB) at the University of Arizona (IRB protocol #141545697R03).

### 2.2. Isolation and Expansion of MSCs

Cells from adipose samples were isolated by enzymatic digestion. Briefly, 20 mL of adipose tissue was placed in a 50 mL Falcon tube and washed vigorously four times with 10 mL PBS. Cells in the wash fraction were also retained. The fatty tissue was treated with an equal volume of 0.2% collagenase type IV at 37 °C for 30 min (Sigma (Marlborough, MA, USA), research grade). Then, 20 mL of complete or expansion medium was added to the digested tissue to neutralize the collagenase, and the sample was washed by centrifugation twice, passed through a 100 μm filter to remove cell clumps, and then centrifuged at 150 *g* for 10 min to obtain the adipose-derived cells. The cells from both the wash and the digested fractions were suspended in complete or expansion medium and counted using Acridine Orange (total cell counts) and Propidium Iodine (viability), using a Cellometer. A total of 0.2–1 × 10^5^ viable cells were cultured in a 25 cm^2^ culture flask. After 3–4 days, the unattached cells were depleted by replacing the medium with fresh medium. Following this, the medium was changed twice a week. At 80–90% confluency, the cells were harvested with trypsin–EDTA. Complete medium (Minimal Essential Medium; Thermo Scientific, Waltham, MA, USA; 500 mL) was supplemented with 10% fetal bovine serum (FBS; Hyclone (Logan, UT, USA) or Atlanta Biologicals (Flowery Branch, GA, USA)) and 1% each of non-essential amino acids, sodium pyruvate, glutamine, and streptomycin/penicillin solution (Hyclone) as the baseline medium.

### 2.3. Expansion Media

Adipose-derived MSCs from the same donor were split into replicate cultures and grown in either standard FBS-containing medium or one of the synthetic media, starting at day 0 d0 (P0) of culture. At the indicated time points, the cultures were harvested and analyzed for total cell numbers, viable cells, and surface phenotype. The synthetic media utilized in the MSC expansion cultures were StemMACS MSC Expansion Media (Miltenyi Biotec), PLTMax Human Platelet Lysate (Sigma-Aldrich), and MesenCult-hPL media (Stemcell Technologies). All synthetic media were utilized according to the manufacturers’ instructions.

### 2.4. Cell Surface Antigen Profile of Adipose-Derived Cells

Cell surface protein expression was evaluated by flow cytometry. The cells were harvested at the indicated times by treatment with 0.05% trypsin–EDTA (5 min, 37 °C), pelleted by centrifugation, re-suspended in PBS, and counted. A total of 1 × 10^5^ cells were incubated with the following primary antibodies: anti-CD45, -CD73, -CD90, and -CD105 conjugated with FITC (BD Pharmingen, San Diego, CA, USA), phycoerythrin (PE, BD), allophycocyanin (APC, Biolegend (San Diego, CA, USA)), and Alexa Fluor 700 (AF-700, Biolegend), respectively, for 30 min at 4 °C. The samples were analyzed using an LSR II flow cytometer (BD, USA) and FACS DIVA software (BD Biosciences, Franklin Lakes, NJ, USA). Unstained cells were used to establish flow cytometer settings. Debris and auto-fluorescence were removed using forward scatter. At least 1 × 10^4^ gated events were used for each analysis.

### 2.5. Osteoblast and Adipocyte Differentiation 

To assess the differentiation potential of the adipose-derived MSCs, two types of directed differentiation were examined: osteogenic and adipogenic.

For osteogenic differentiation, MSCs were plated in 12-well plates at a final cell density of 5 × 10^3^ cells/cm^2^ in complete medium. After 24–48 h when the cells were 80–90% confluent, the complete medium was replaced with osteogenic differentiation medium (AdvanceSTEM (Bangkok, Thailand) osteogenic differentiation medium, catalog no. SH30881.02; Thermo Scientific) supplemented with 10% AdvanceSTEM stem cell growth supplement (catalog no. SH30878.02; Thermo Scientific). The medium was changed twice a week for 3 weeks. The osteogenically induced cells were stained with Alizarin Red S (C. I. 58005). Briefly, after removal of culture medium, the cells were washed with PBS and fixed with formalin. All wells were stained with Alizarin Red S (C. I. 58005) for 45 min at 37 °C. The wells were washed with distilled water, and the red stained calcium deposits were reviewed under a light microscope.

For adipogenic differentiation, MSCs were seeded in 12-well plates at a final cell density of 5 × 10^3^ cells/cm^2^ and propagated in complete growth medium—either FBS, MesenCult hPL, StemMACS, or PLTMax. Differentiation was initiated 48 h later, which was designated as day 0, using adipogenic induction medium (AdvanceSTEM adipogenic differentiation medium, catalog no. SH30886.02; Thermo Scientific) supplemented with 10% AdvanceSTEM stem cell growth supplement (catalog no. SH30878.02; Thermo Scientific). The medium was changed every 3–4 days thereafter, and the experiments were terminated after 3 weeks. After 3 weeks, the adipose differentiated cells were stained with a commercially available oil O red staining kit (IHC World, Woodstock, MD, USA) to visualize lipid droplets. Briefly, the culture medium was discarded, and the cells were washed three times with PBS, fixed with formalin, washed with distilled water, and treated with 60% isopropanol for 5 min. The pre-warmed oil O red solution was added in excess to each well, and the plate was incubated at 37 °C for 45 min. Each well was washed with distilled water. Finally, the cells were counterstained with a hematoxylin solution for 1 min. The lipid bodies were observed under light microscopy in at least eight non-overlapping fields.

## 3. Results

Preliminary experiments showed that each of the xeno-free media could sustain cell expansion, viability, and identity when MSCs were switched from FBS-containing medium into each of the synthetic media (data not shown). Therefore, a more detailed analysis was performed for adipose-derived MSCs grown in each of the synthetic media for a period of 5 weeks. MSCs were analyzed weekly for cell viability, cell expansion, and phenotype. Three independent adipose tissue donors were utilized for the experiments.

The synthetic media did differ significantly from one another when compared to standard FBS-containing cultures in terms of cell expansion. As shown in Table 1, when MSCs were grown in FBS-containing cultures, it generally took 4 weeks for any significant cell expansion to be observed (all values are represented as fold-increase values, obtained by normalizing the number of cells counted at the end of each week to the number of cells counted at the end of the first week, arbitrarily set to 1). That is, by week 4, the MSCs grown in FBS-containing medium had expanded to 8.6 times the initiating cell dose by the end of the first week. Each of the synthetic media however, displayed significant cell expansion by the second week of culture. The cells in the StemMACS and hPL media grew faster than the cells in the PLTMax medium, while all synthetic media supported greater cell expansion than the FBS-containing medium. Overall, the hPL medium supported larger cell growth than either the StemMACS or the PLTMax media at each of the time points (with the possible exception of week 4 for the StemMACS cultures). As shown in Table 2, each of the synthetic media was capable of maintaining MSCs from multiple donors at high viability over a period of 5 weeks. No significant difference was found in cell viability at any of the time points with any of the media. Once the cultures were established with any of the media after the initial first week of culture, it was unusual to observe cell viabilities less than 80%.

To determine if cell expansion in any of the synthetic media had an effect on MSC cell surface antigen expression, cells from each of the cultures were examined for surface phenotype after 3 and 5 weeks in expansion cultures. Initial cell cultures were CD45-negative and remained so throughout the cultures, while CD34 expression was less than 1% at P0 and undetectable thereafter. As shown in Table 3, MSCs obtained from each of the cultures at weeks 3 and 5 were uniformly positive for the CD90 marker often used as an MSC marker (less than 10% change at any time point in any of the media including FBS-containing medium). However, MSCs grown in hPL medium decreased their expression of CD73 at week 3 (decrease from 100% positive to 65% positive), which then recovered to initial week 1 levels by week 5 (change from 65% positive to 100% positive). MSCs expanded in any of the other media maintained a uniform CD73 expression at each time point, between 91% and 100% positive. CD105 expression varied the greatest in culture, regardless of which medium was used for cell expansion. Culture of MSCs in FBS-containing medium resulted in a 24% loss of expression by week 5 in culture. Culture in hPL medium resulted in an even greater loss of expression (84%), while MSC expansion in StemMACS medium resulted in a small increase in expression (by 16%) and in PLTMax media resulted in a 21% upregulation of CD105 expression. 

**Figure 1:**
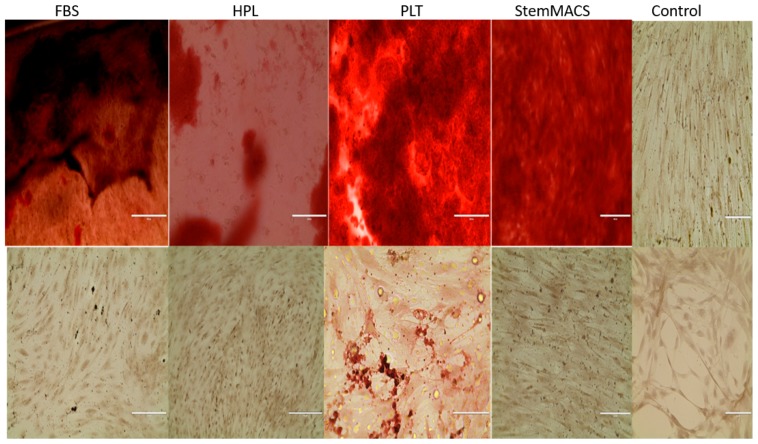
Directed differentiation of MSC expanded in synthetic media. MSC were expanded in each of the synthetic media as described in Table 1. At the end of 4 weeks of culture, the cells were harvested, counted, and re-plated in differentiation media for a subsequent 3 weeks. At that time, the cells were analyzed either for osteogenic (**top panel**) or adipogenic differentiation (**bottom panel**) by histology. Magnification for all photographs is 10×.

Finally, MSCs expanded for 5 weeks in each of the synthetic media were analyzed for the ability to undergo directed differentiation typical of MSCs. At the termination of the expansion cultures, MSCs were placed in adipocyte and osteocyte differentiation cultures for an additional 21 days. It was observed that expanded MSCs from each of the synthetic media cultures were capable of differentiating into both adipocytes (not shown) and osteocytes (Figure 1). 

## 4. Discussion

MSCs, which can be isolated from different human tissues, are a preferred cell source in the field of regenerative medicine. MSCs show self-renewal capability in association with differentiation potential along mesenchymal and non-mesenchymal lineages [6]. These features make MSCs ideal therapeutic candidates for cell-based therapies and genetic engineering. Significant decreases in available MSC cell numbers, along with a reduction in the proliferative and differentiation capability due to in vitro and in vivo aging [7,8], make bone marrow a less preferred source of MSC. Attention is now being paid to alternative sources of MSC that are readily available, need less invasive procedures to be obtained, and have a higher proliferative and differentiation potential, such as adipose tissue-derived MSC. Potential health risks associated with the use of stem cells grown in animal-derived media, such as those containing FBS, have delayed the advancement of stem cell therapies. The development of animal-free/xeno-free synthetic media is a crucial step in increasing the clinical use of stem cells, as stem cells are generally limited in number when first harvested.

There has been a limited number of studies in the literature comparing different synthetic media to one another as well as to FBS-containing media, with mixed results concerning MSC expansion [9,10,11]. Riis et al. (2016) found that hPL provided the best results in terms of cell expansion, similar to our own findings, while MSC cultured in StemPro (Invitrogen, Carlsbad, CA, USA) did not proliferate as well or for more than several passages. In contrast, however, Lindroos et al. reported in 2009 that adipose-derived MSC proliferated as well in StemPro as in FBS-containing media. In addition, they observed that CellGro (CellGenix, Freiburg, Germany) synthetic media provided superior cell expansion results to those obtained with cells cultured in FBS-containing media. Finally, Dessels et al. (2016) reported that either hPL or platelet-rich plasma (PRP) could comparably substitute for FBS in cell expansion media. The reason for these discrepancies is unknown but may be due to the development of newer synthetic media. However, none of these past studies examined the effects of the synthetic media utilized in the current study.

In the present study, we examined the ability of different commercially available synthetic, xeno-free media to support MSC expansion, as this is needed for many regenerative medicine applications. At time points of up to five weeks in culture, we analyzed the effects of such media on cell numbers, cell viability, cell surface phenotype, and directed differentiation. Compared to the FBS-containing medium, the xeno-free media PLTMax, StemMACS, and Mesencult-hPL, showed to have the capability of expanding MSCs in culture at a greater rate, while maintaining high cell viability and typical MSC phenotypic and functional characteristics. MSC grown in each of the xeno-free media were at least 85% viable after four weeks in culture, displayed a CD73+ CD90+ CD105+ phenotype, and were able to differentiate into both adipogenic and osteogenic lineages.

In each of the media examined, cells took approximately one week to reach confluency in a T-25 culture flask, so to be passaged for subsequent expansion. Once confluency was reached however, it was observed that the cells in the xeno-free media expanded at a much greater rate than the cells grown in the FBS-containing medium. Cells grown in the synthetic media maintained high levels of cell viability and remained healthy, even compared to the FBS cultures. Interestingly, when observed under a microscope, while in culture, it was found that the MSCs grown in FBS cultures seemed larger than the cells grown in each of the xeno-free medias at later time points, i.e., they displayed morphological characteristics associated with cell stress and senescence. All MSCs grown in xeno-free media seemed to be similar in size to one another. Whether this morphological characteristic is related to cell expansion capacity is not known, but it could affect the number of consumables needed to attain similar cell numbers. 

As a technical note, MSCs grown in hPL synthetic medium required no medium change or other intervention for the first two weeks of culture, while the other media required medium changes approximately every 48 h. Thus, the hPL cultures required less technician time, which could greatly reduce the overall costs of such endeavors. MSCs cultured in the hPL and PLTMax media consistently produced substantially more cells than the FBS cultures. However, when the StemMACs cultures were allowed to reach total confluency, passaging the cells was difficult and inefficient, as they often clumped together and were difficult to re-plate and grow. Thus, in terms of methodological ease and reduced overall costs, the use of either hPL or PLTMax would be preferable for clinical applications.

In conclusion, each of the synthetic media tested were suitable replacements for the standard use of FBS-containing expansion media. Importantly, long-term expansion in any of the synthetic media had no significant effect on MSC function, as evidenced by the cells' ability to differentiate into adipocytes and osteocytes. This observation should not be overlooked in terms of its importance, as simply obtaining large numbers of mesenchymal cells that are not functional would be useless for regenerative medicine applications (as well as terribly expensive).

## Figures and Tables

**Table 1:** Mesenchymal stromal cells (MSC) cell counts after long-term expansion in synthetic media.

Media	Week 1	Week 2	Week 3	Week 4
FBS	1.0	1.7	1.9	8.6
StemMACS	1.0	9.5	6.3	26.8
HPL	1.0	26.9	17.7	33.4
PLT Max	1.0	2.4	3.7	3.4

MSCs were plated for one week in fetal bovine serum (FBS)-containing medium after adipose tissue digestion as described, harvested, and then re-plated in each of the synthetic media. The cells were harvested and counted at the end of each subsequent week. The data are presented as fold-increase in total cell counts and are normalized to viable cell counts obtained at the end of week 1 in culture from three independent experiments.

**Table 2:** MSC cell viability after long-term expansion in synthetic media.

Media	Week 1	Week 2	Week 3	Week 4
FBS	73	91	90	87
StemMACS	73	86	74	85
HPL	73	91	90	89
PLT Max	68	92	77	91

MSCs were plated for one week in FBS-containing medium after adipose tissue digestion as described, harvested, and then re-plated in each of the synthetic media. The cells were harvested, and viability was determined at the end of each subsequent week. The data are presented as the percent viable cells obtained in three independent experiments.

**Table 3:** MSC antigen expression after 3 and 5 weeks of expansion culture in synthetic media.

Media	CD105		% Change	CD73		% Change	CD90		% Change
	Week 3	Week 5		Week 3	Week 5		Week 3	Week 5	
FBS	100	76	−24	100	97	−3	100	93	−7
StemMACS	100	16	−84	65	100	+54	99	92	−7
HPL	83	99	+19	100	100	+0.2	100	100	0
PLT Max	77	98	+27	91	100	0	100	100	0

MSCs were cultured as described in Table 1 and Table 2. At the end of weeks 3 and 5 of culture, MSCs were analyzed for phenotype by flow cytometry, as described. The data are presented as percent positive cells for each antigen and change in expression from one time point to another.
